# Supplementary figures and images for: Perilipin-related protein regulates lipid metabolism in C. elegans
Source: PeerJ. 2015 Sep 1;3:e1213. doi: 10.7717/peerj.1213 (PMC4562238; doi:10.7717/peerj.1213)

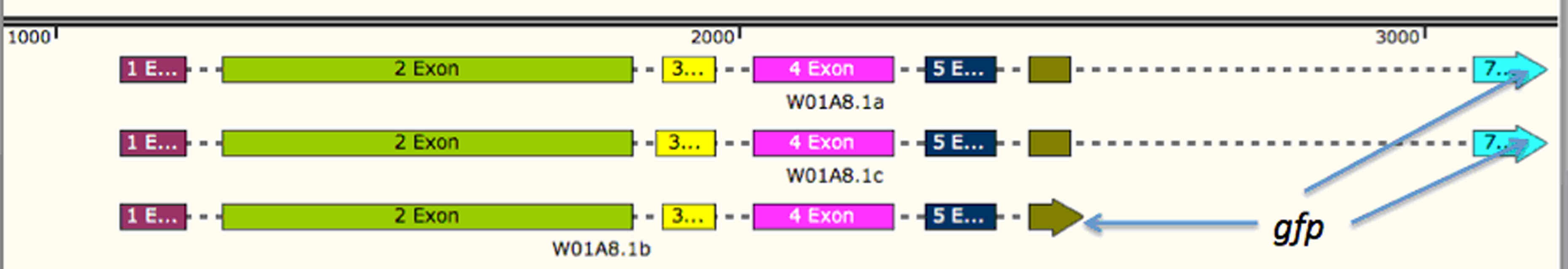

Supplement: Figure S1 — Three different protein isoforms are expressed from the W01A8.1 gene. The gene and the expressed proteins are denominated as W01A8.1 a, b and c (as accessed in Wormbase WS 246 on March 14, 2015). The gene coding for GFP is inserted before the STOP codon in the 7th exon (marked as W01A8.1a/c::gfp) or in the 6th exon of isoform b (marked as W01A8.1b::gfp). The transgene based on the first construct (covering the upper two isoforms a and c) may lead to the expression of both isoforms a or c fused to GFP dependent on the cellular context and is likely to lead also to overexpression of untagged isoform b from the extra-chromosomal array. The genomic map was designed using SnapGene software (from GSL Biotech; available at snapgene.com). [file peerj-03-1213-s001.jpg]

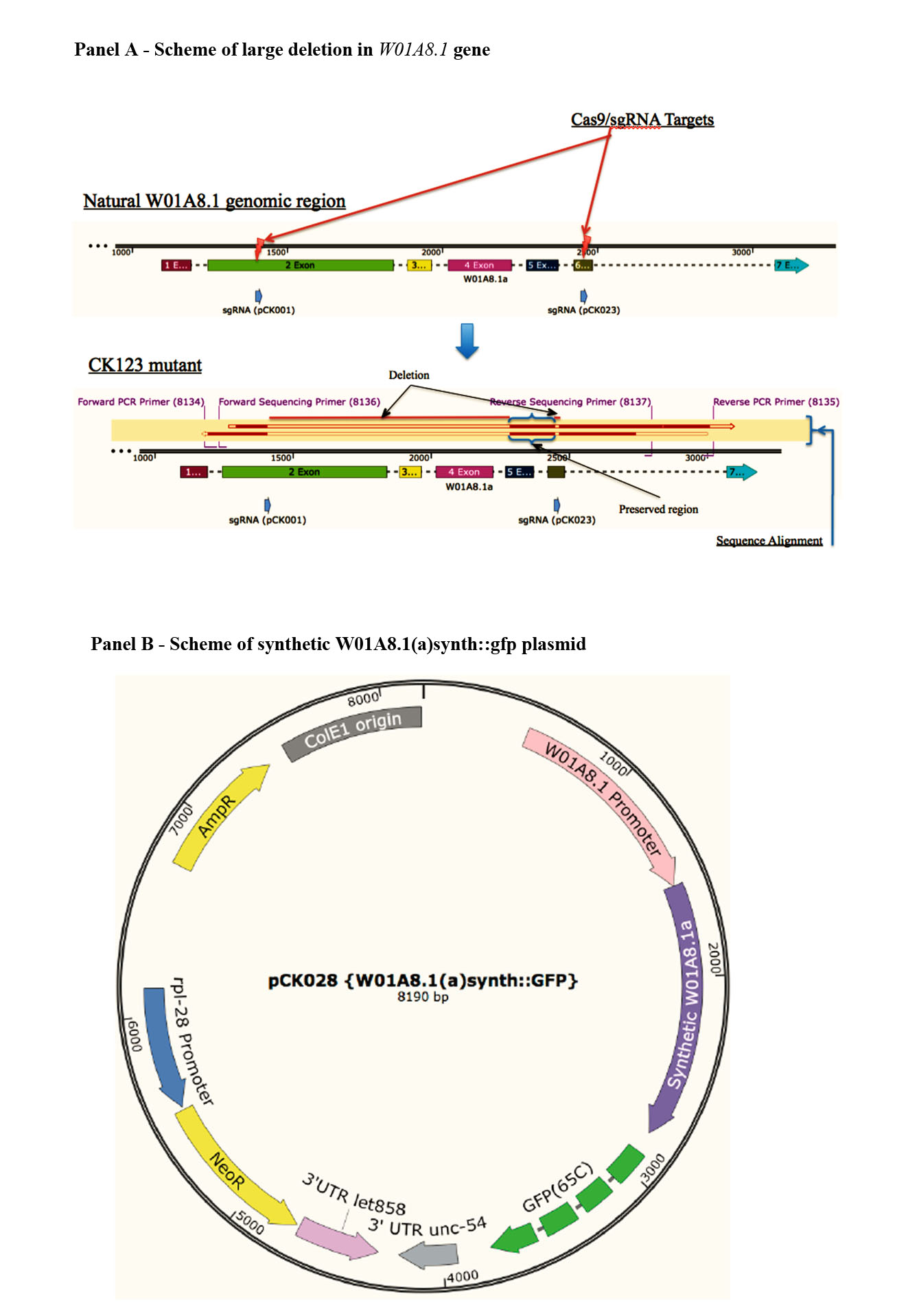

Supplement: Figure S2 — (A) shows the genomic region of W01A8.1 gene being targeted by CRISPR/Cas9 sgRNAs at two marked positions +. After selection, the mutant CK123 (bottom) was generated and the genomic segment between primers #8134 and #8135 was PCR amplified and the resulting amplicon was sequenced from both directions using nested sequencing primers #8136 and #8137. It is clear from the sequencing alignment that a significant portion of the gene was disrupted while a small sequence, between the two sgRNA sites, was preserved. (B) represents the extra-chromosomal array used for selection with the CRISPR/Cas9 based deletion shown in (A). The plasmid is carrying a synthetic version of W01A8.1a fused to gfp and was used as a potential balancer if loss of W01A8.1 would be lethal. The plasmid was injected together with mCherry selection markers and CRISPR/Cas9 sgRNA plasmids.The sequence alignment and graphics were designed using SnapGene software. [file peerj-03-1213-s002.jpg]

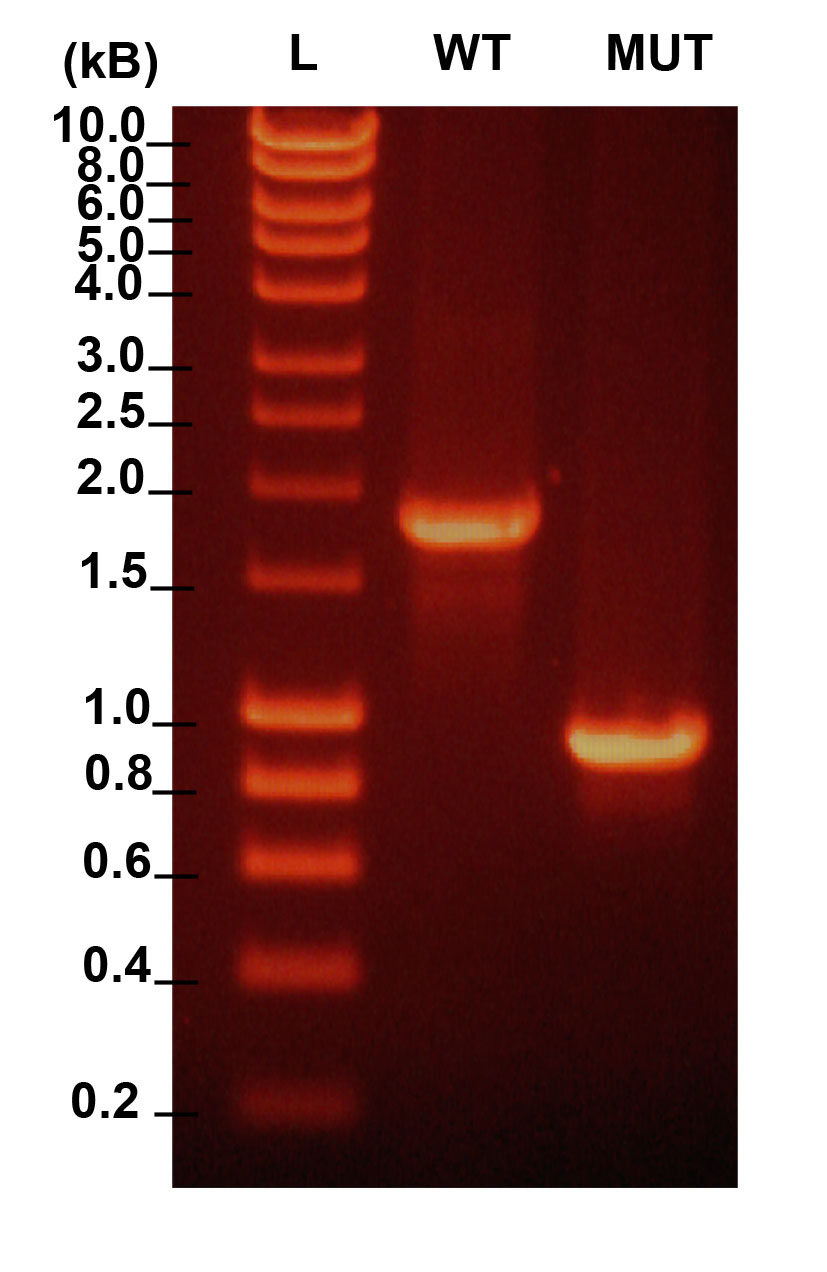

Supplement: Figure S3 — Analysis of W01A8.1 gene by PCR in wild type animals and animals subjected to CRISPR/Cas9 targeted gene deletion. Primers 8134 and 8135 efficiently amplify full length fragment in wild type animals (WT) while mutant animals show homozygous deletion in W01A8.1 gene. [file peerj-03-1213-s003.jpg]

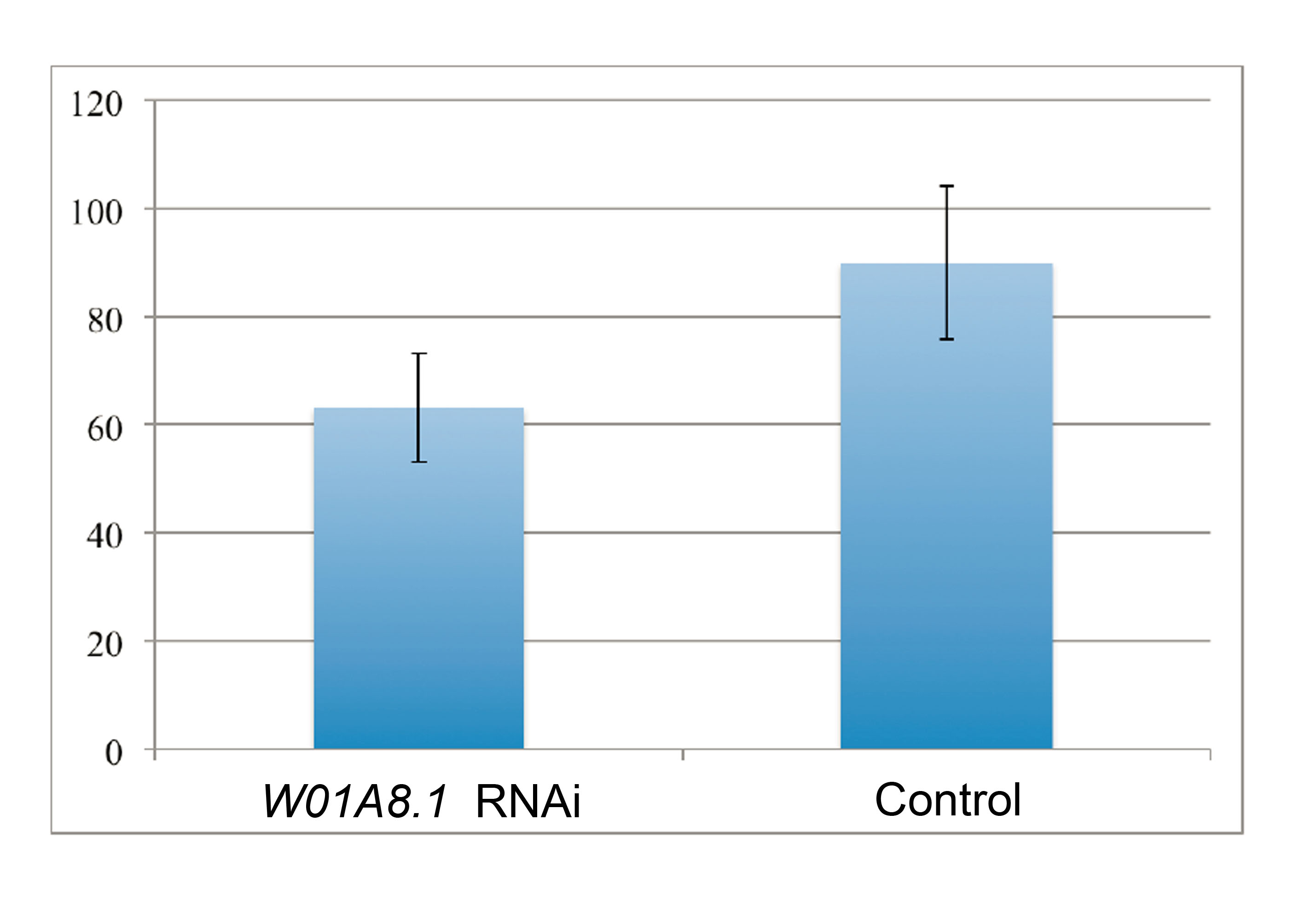

Supplement: Figure S4 — The graph shows knockdown effect of W01A8.1 gene on the amount of progeny compared to controls. Wild type animals were treated with RNAi (dsRNA feeding) directed against W01A8.1 for two generations and the amount of progeny was determined during a 24-hour period per one parent animal (21 parent animals were scored in both categories). The data presented here shows (y-axis) the average number of progeny per parent, n = 1,278 and n = 1,813, respectively, p < 0.05. The SD is indicated. [file peerj-03-1213-s004.jpg]

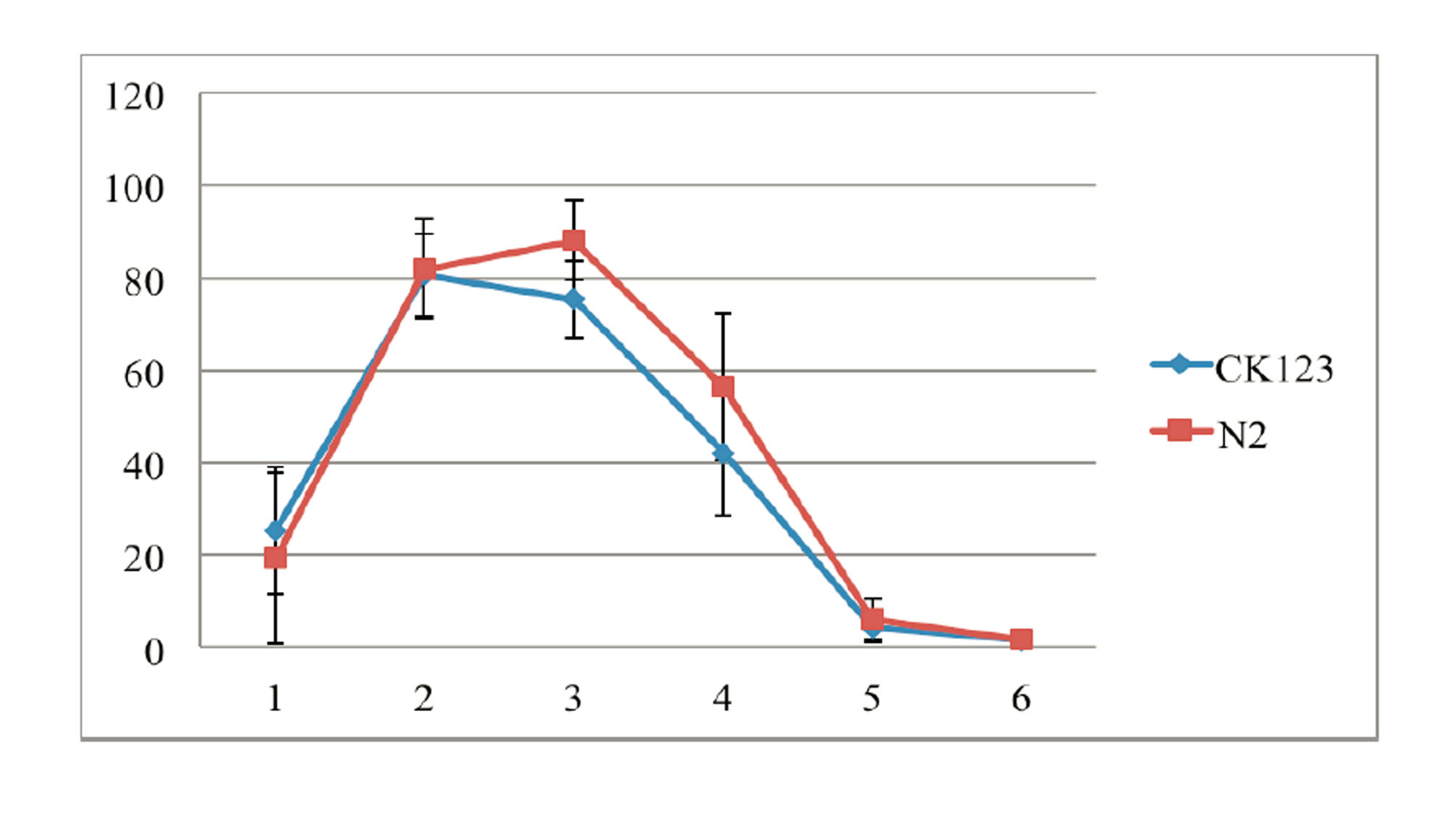

Supplement: Figure S5 — The values show mild statistically significant difference in number of progeny laid by mutant worms CK123 compared to wild type N2 animals during the day 3 (Two Tailed T Test, p = 0.04962). Bars indicate SD. [file peerj-03-1213-s005.jpg]

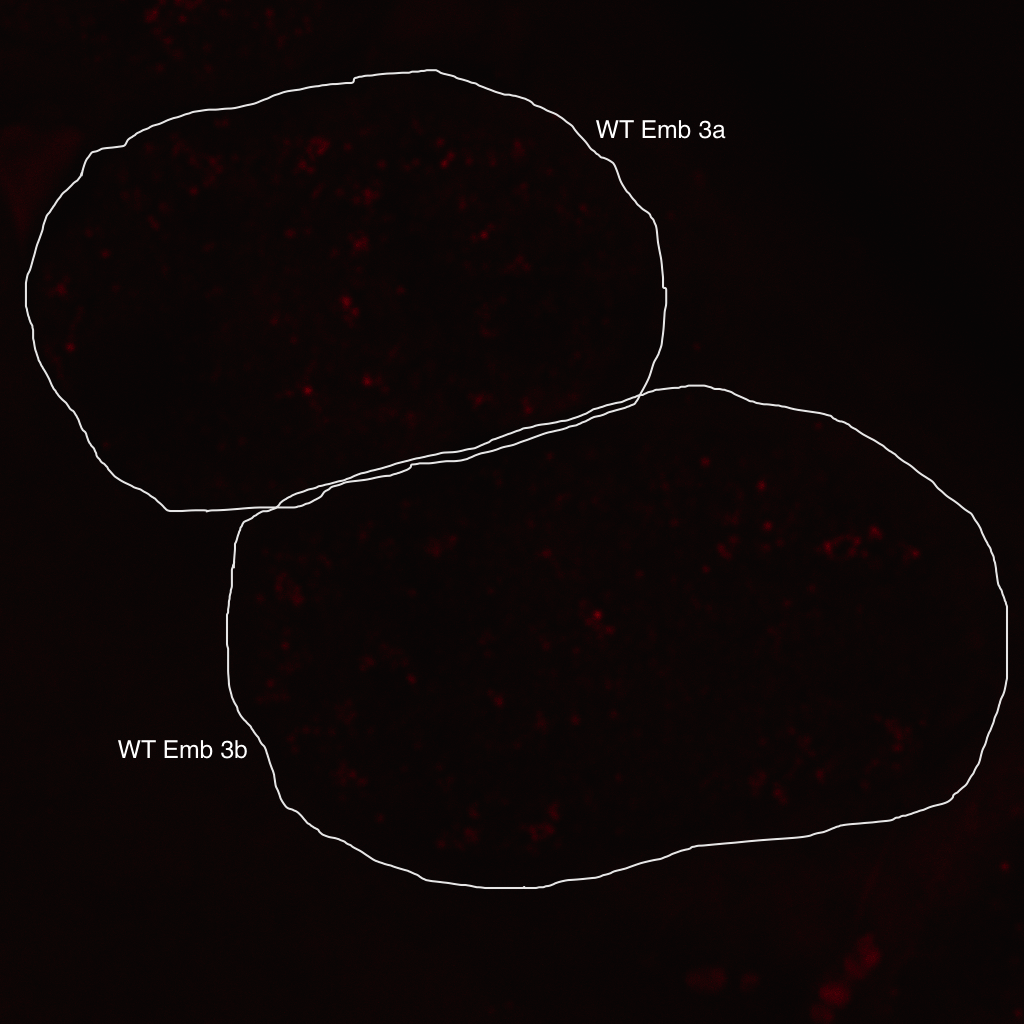

Supplement: Supplemental Information 2 [file peerj-03-1213-s008.zip › Embryos/WT Embryo 3.png]

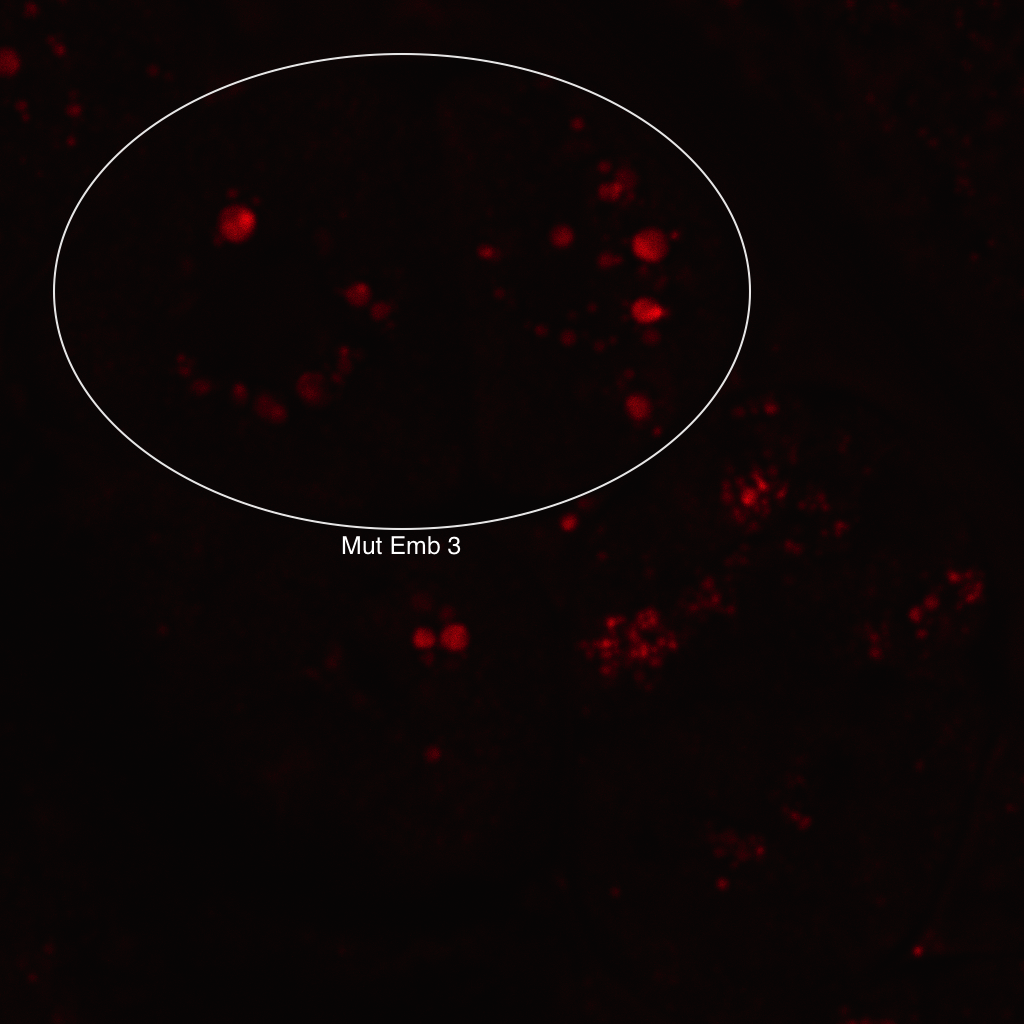

Supplement: Supplemental Information 2 [file peerj-03-1213-s008.zip › Embryos/MUT Embryo 3.png]

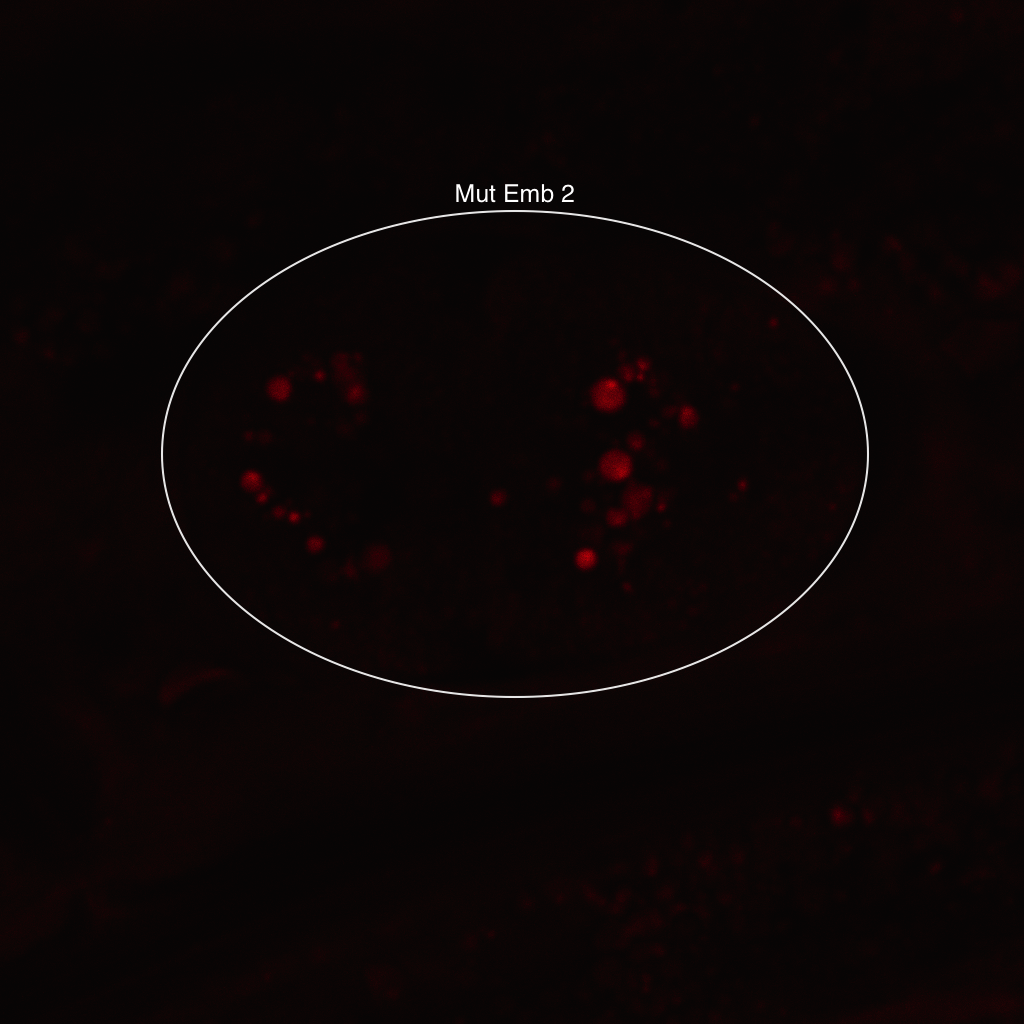

Supplement: Supplemental Information 2 [file peerj-03-1213-s008.zip › Embryos/MUT Embryo 2.png]

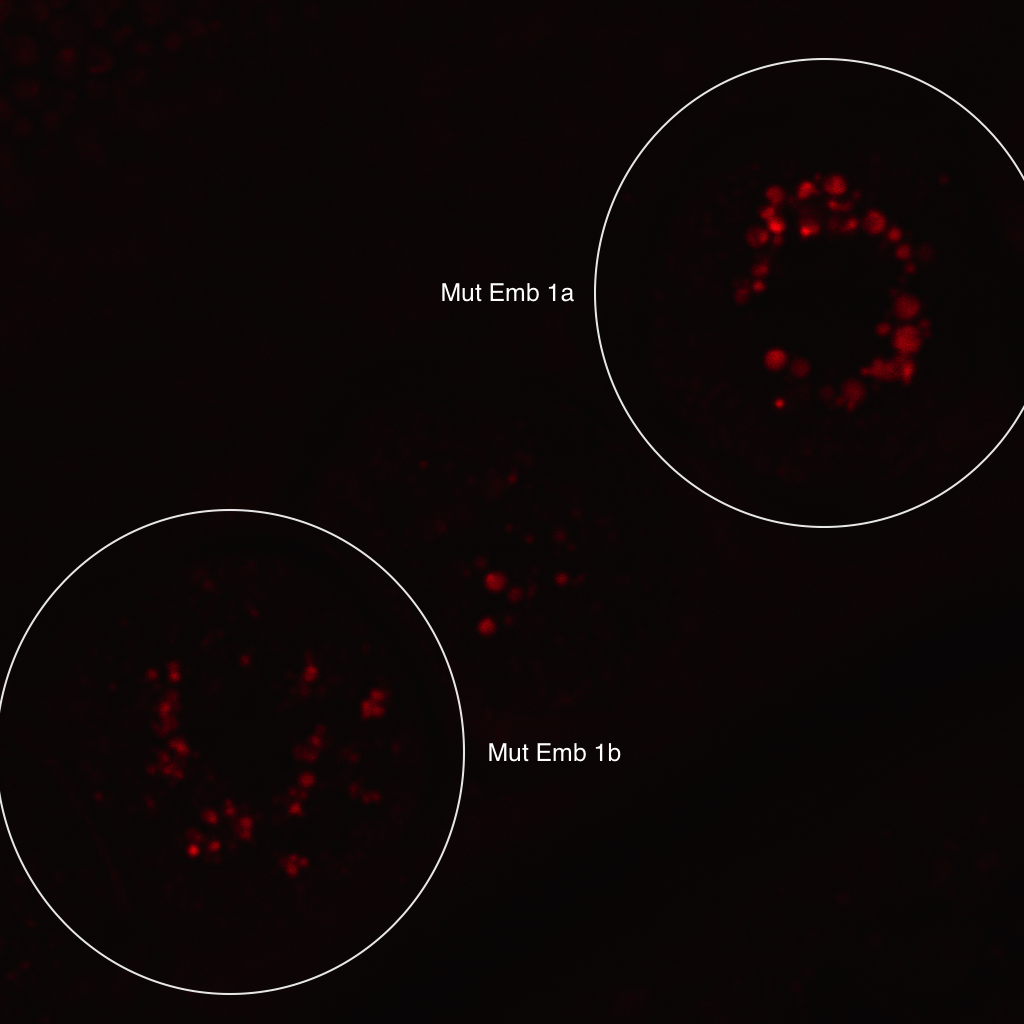

Supplement: Supplemental Information 2 [file peerj-03-1213-s008.zip › Embryos/MUT Embryo 1.png]

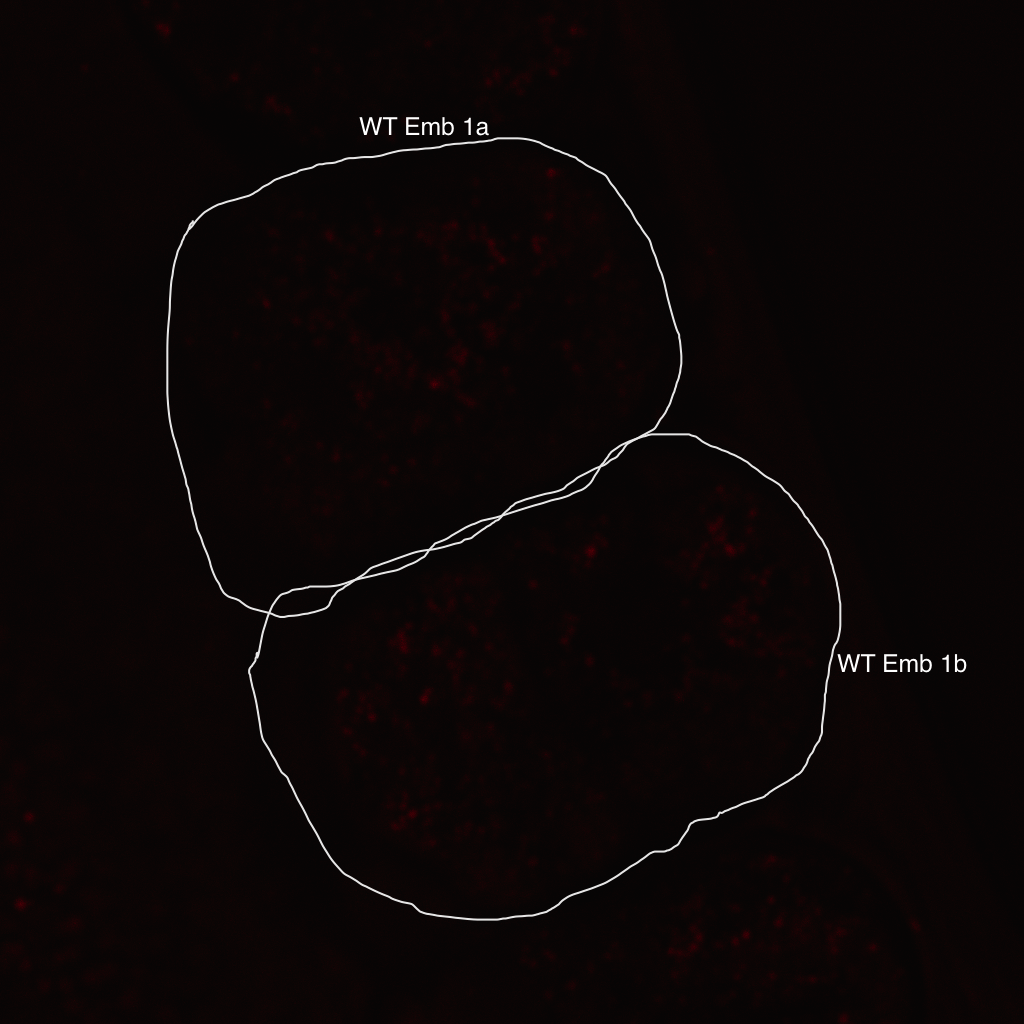

Supplement: Supplemental Information 2 [file peerj-03-1213-s008.zip › Embryos/WT Embryo 1.png]

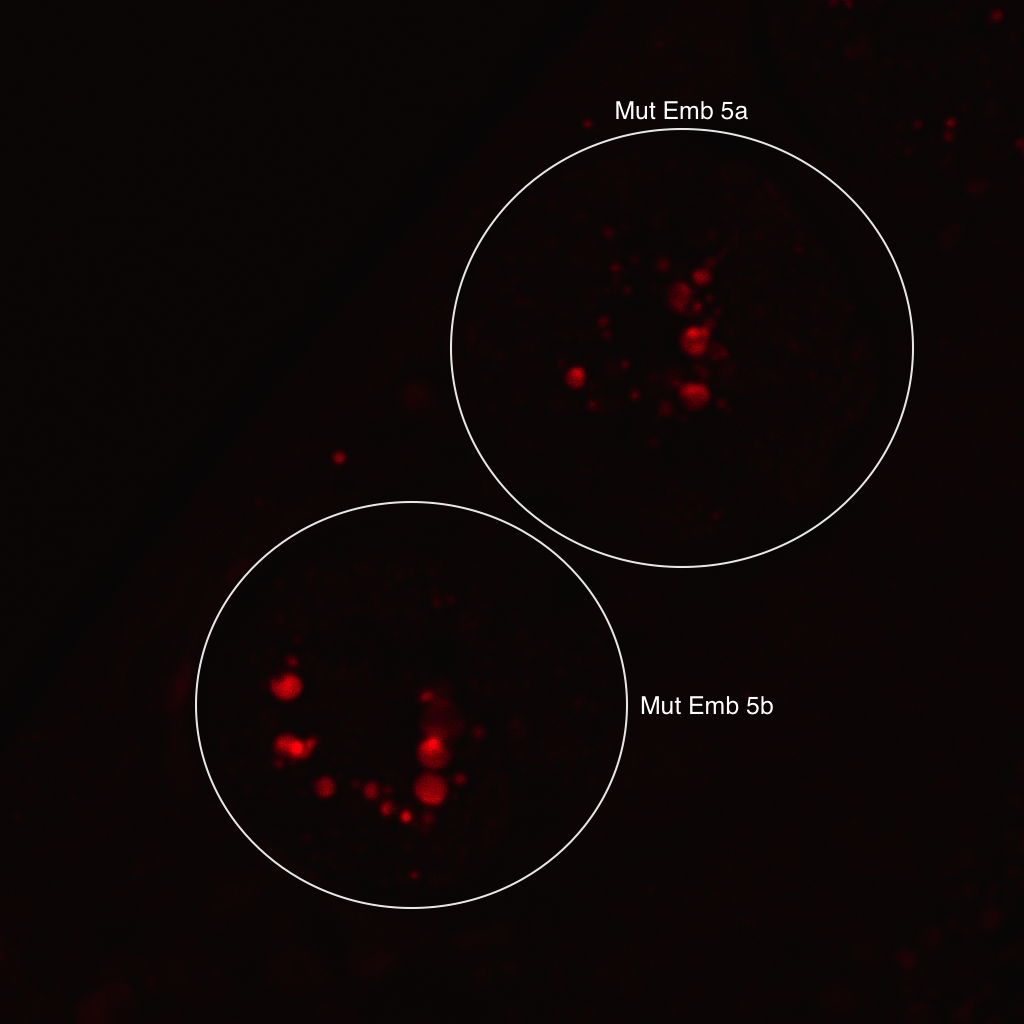

Supplement: Supplemental Information 2 [file peerj-03-1213-s008.zip › Embryos/MUT Embryo 5.png]

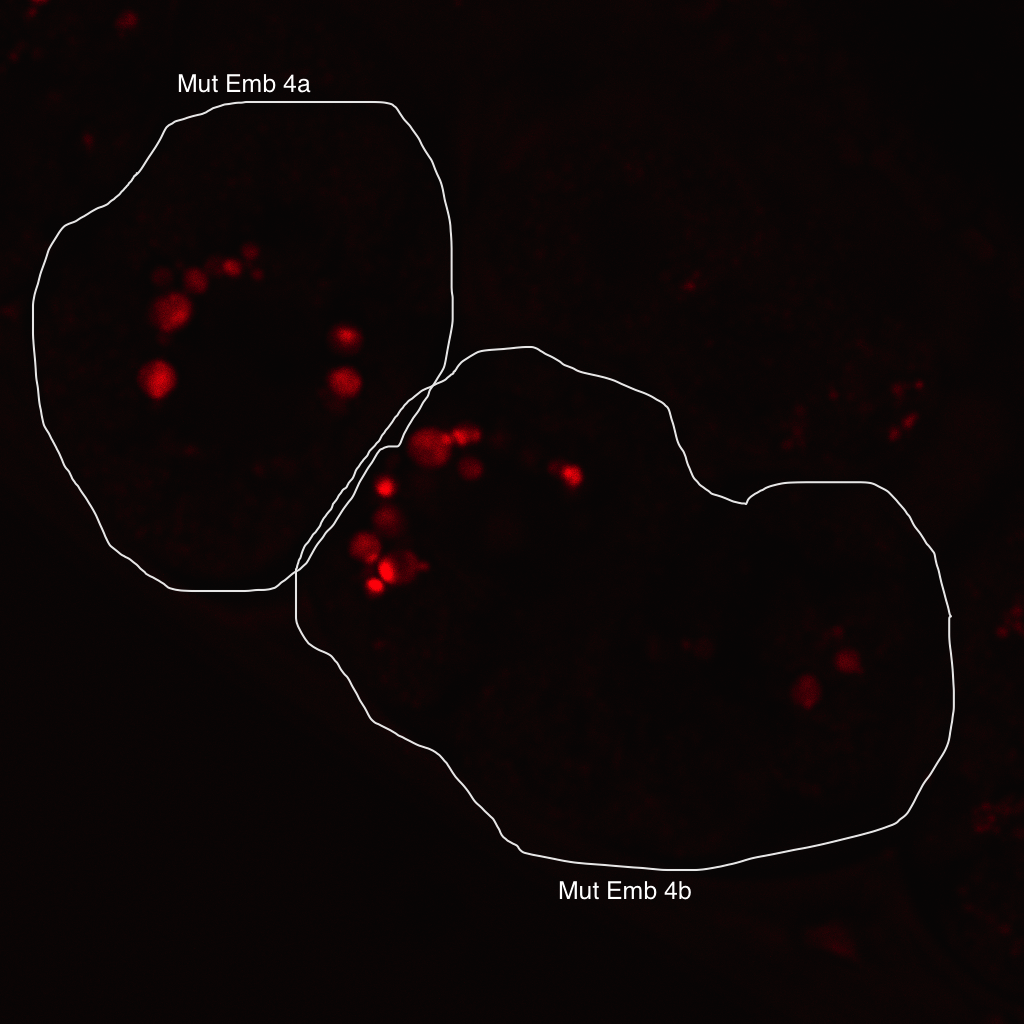

Supplement: Supplemental Information 2 [file peerj-03-1213-s008.zip › Embryos/MUT Embryo 4.png]

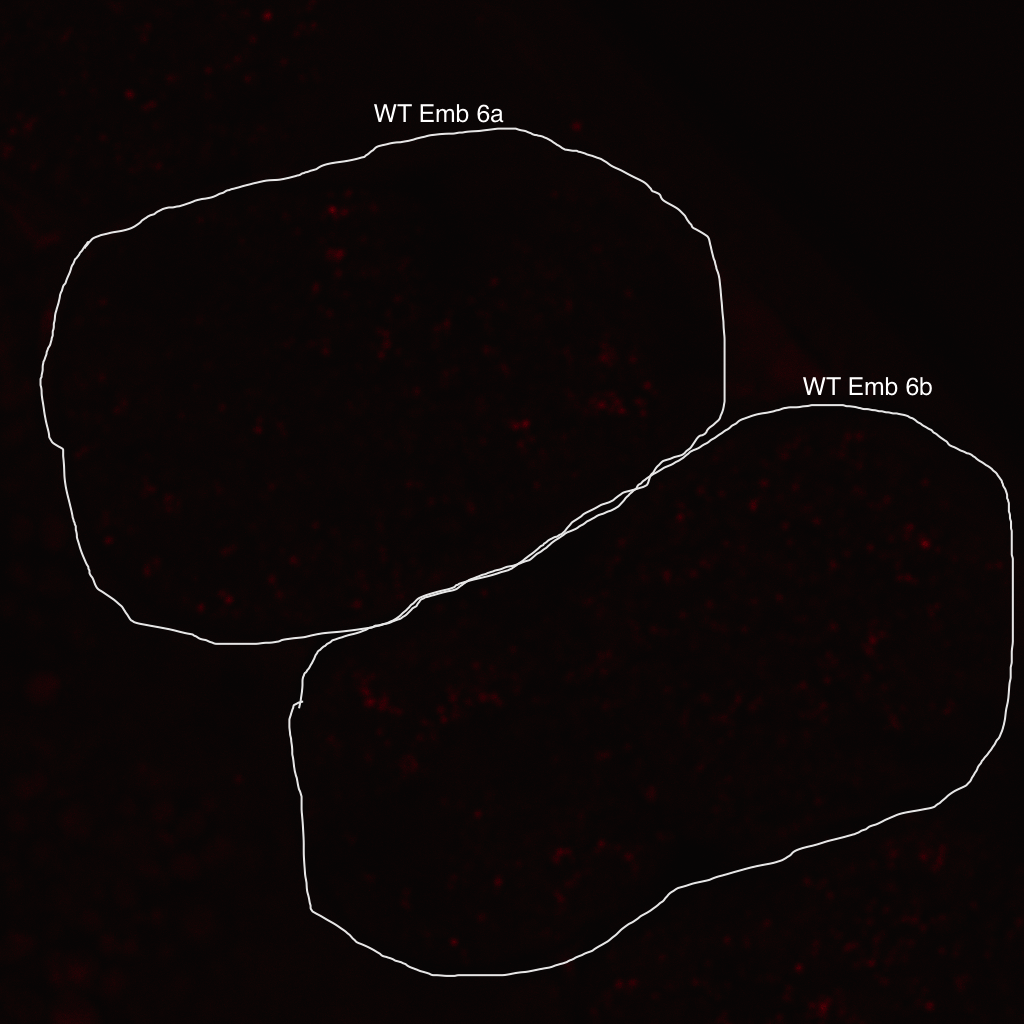

Supplement: Supplemental Information 2 [file peerj-03-1213-s008.zip › Embryos/WT Embryo 6.png]

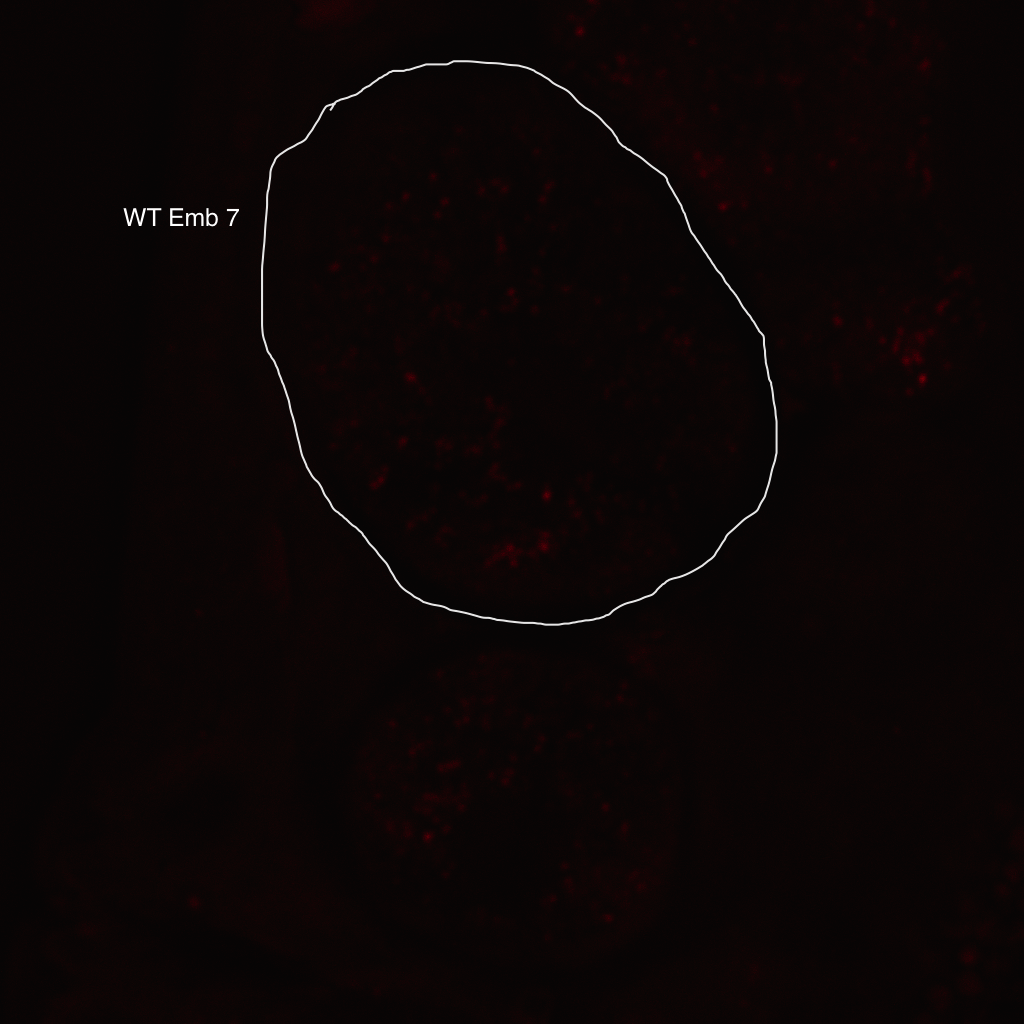

Supplement: Supplemental Information 2 [file peerj-03-1213-s008.zip › Embryos/WT Embryo 7.png]

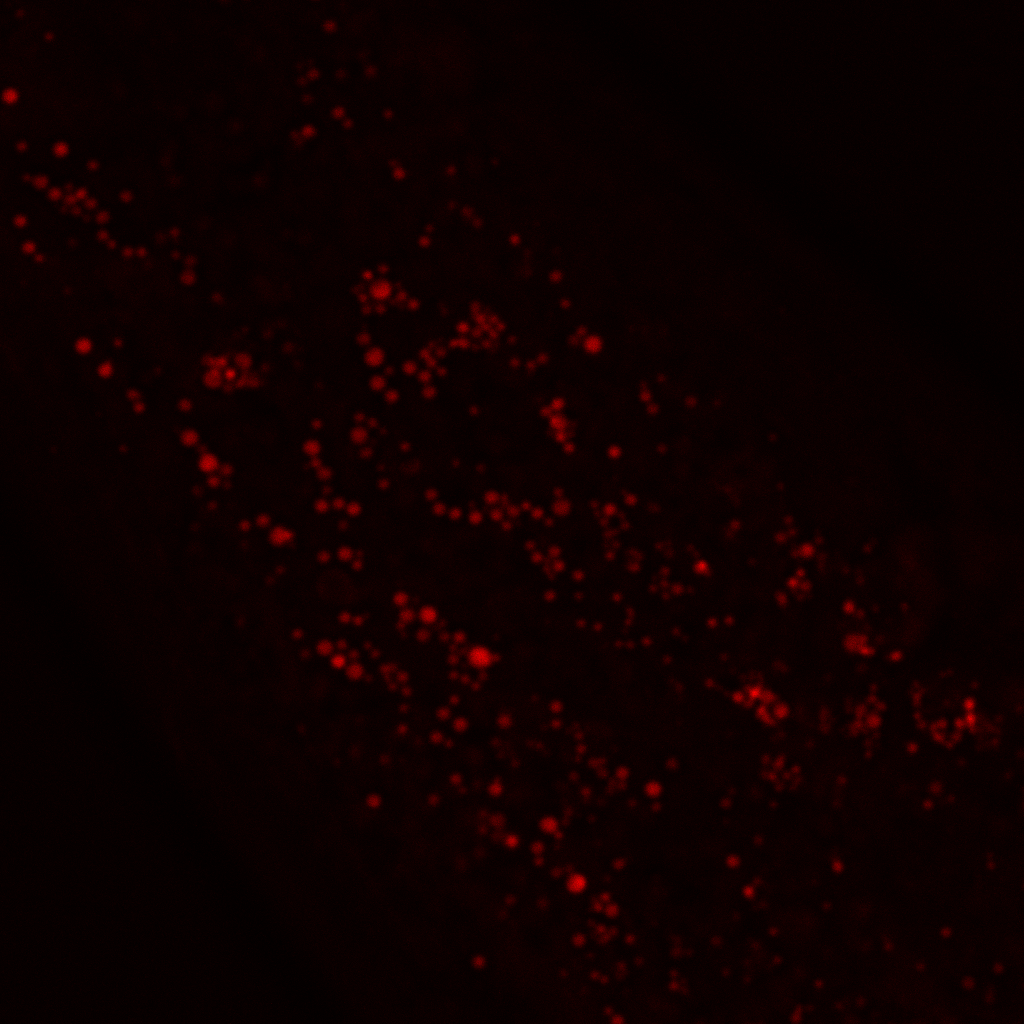

Supplement: Supplemental Information 2 [file peerj-03-1213-s008.zip › Adult/WT Tail 7.png]

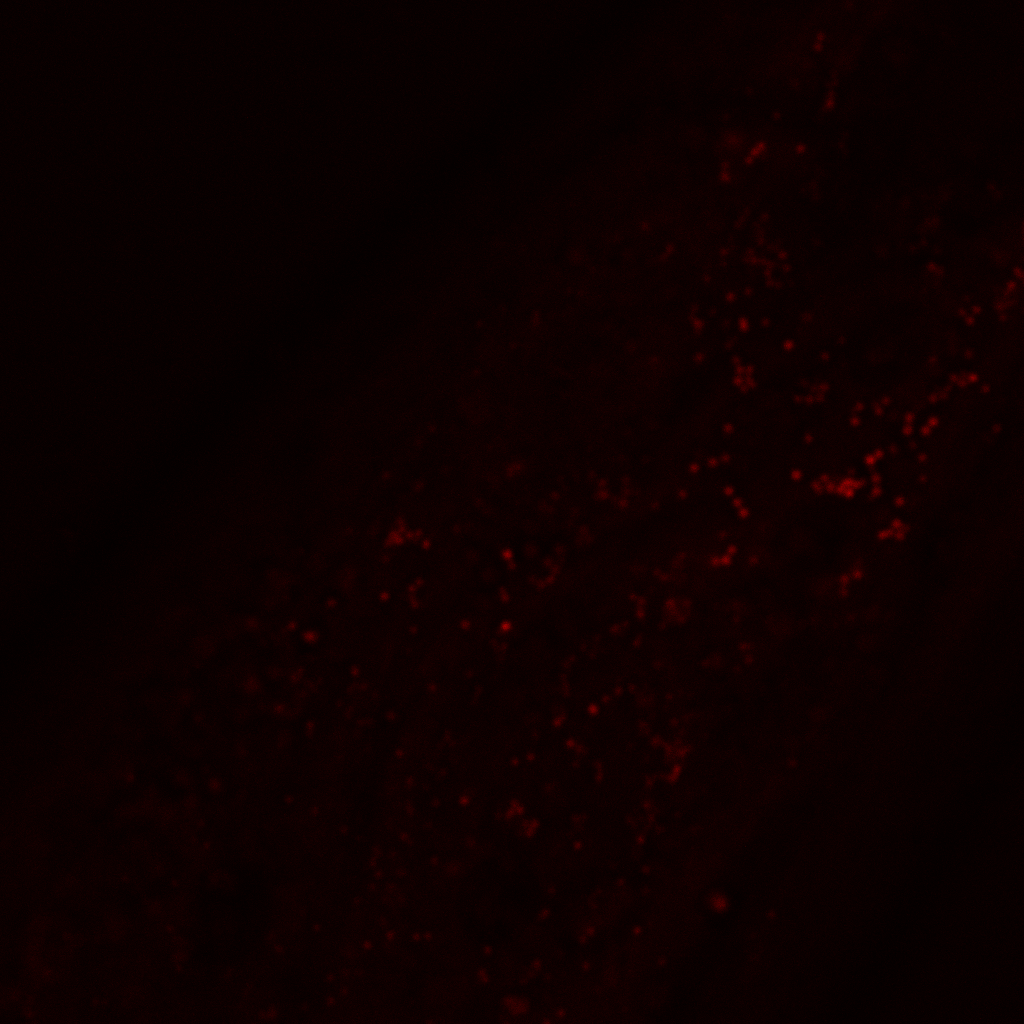

Supplement: Supplemental Information 2 [file peerj-03-1213-s008.zip › Adult/MUT Tail 1.png]

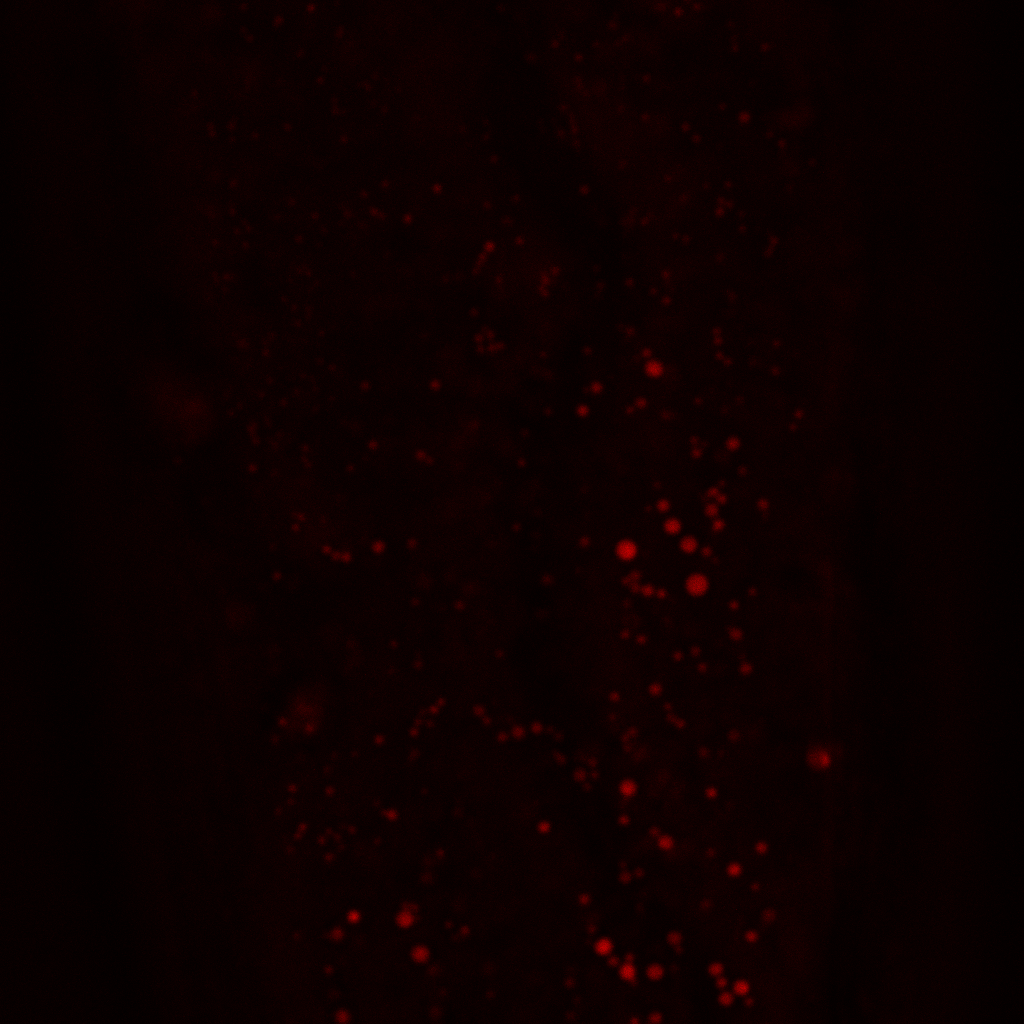

Supplement: Supplemental Information 2 [file peerj-03-1213-s008.zip › Adult/WT Tail 6.png]

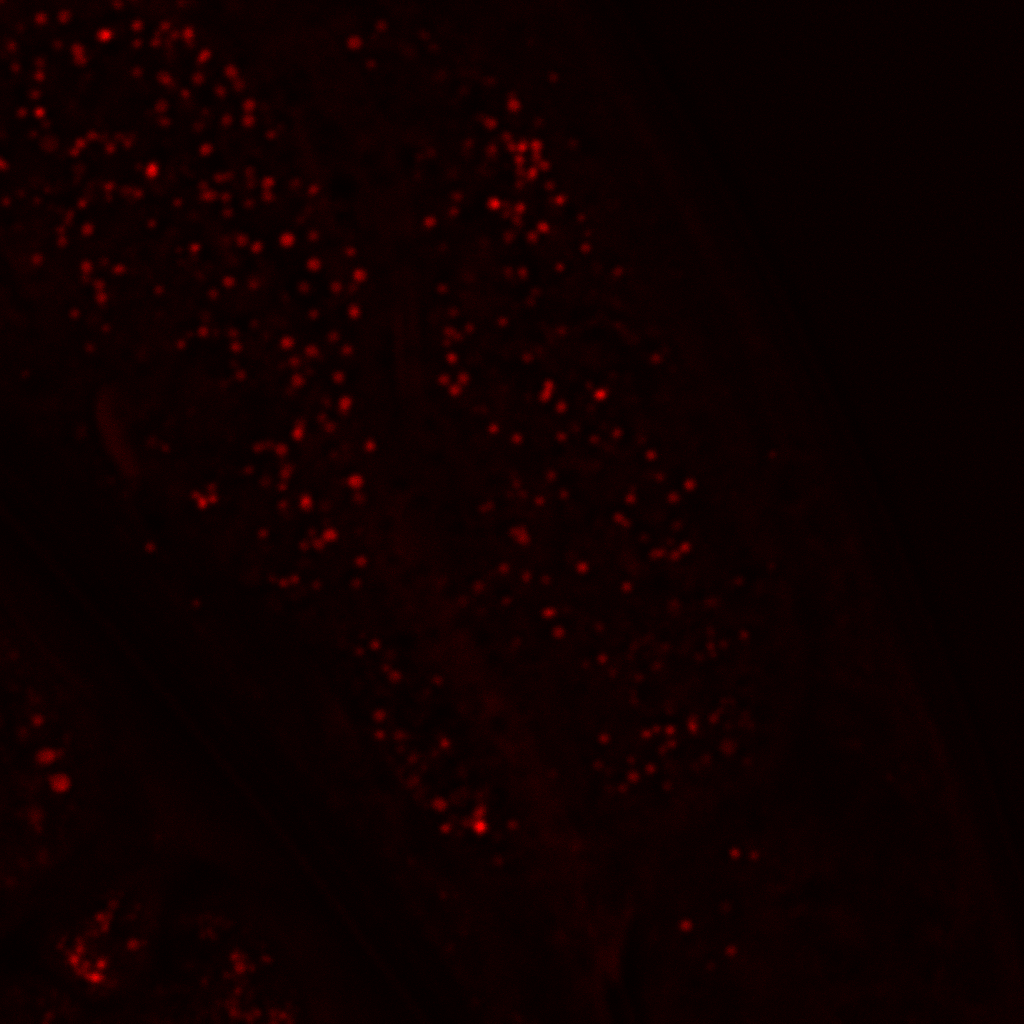

Supplement: Supplemental Information 2 [file peerj-03-1213-s008.zip › Adult/MUT Tail 2.png]

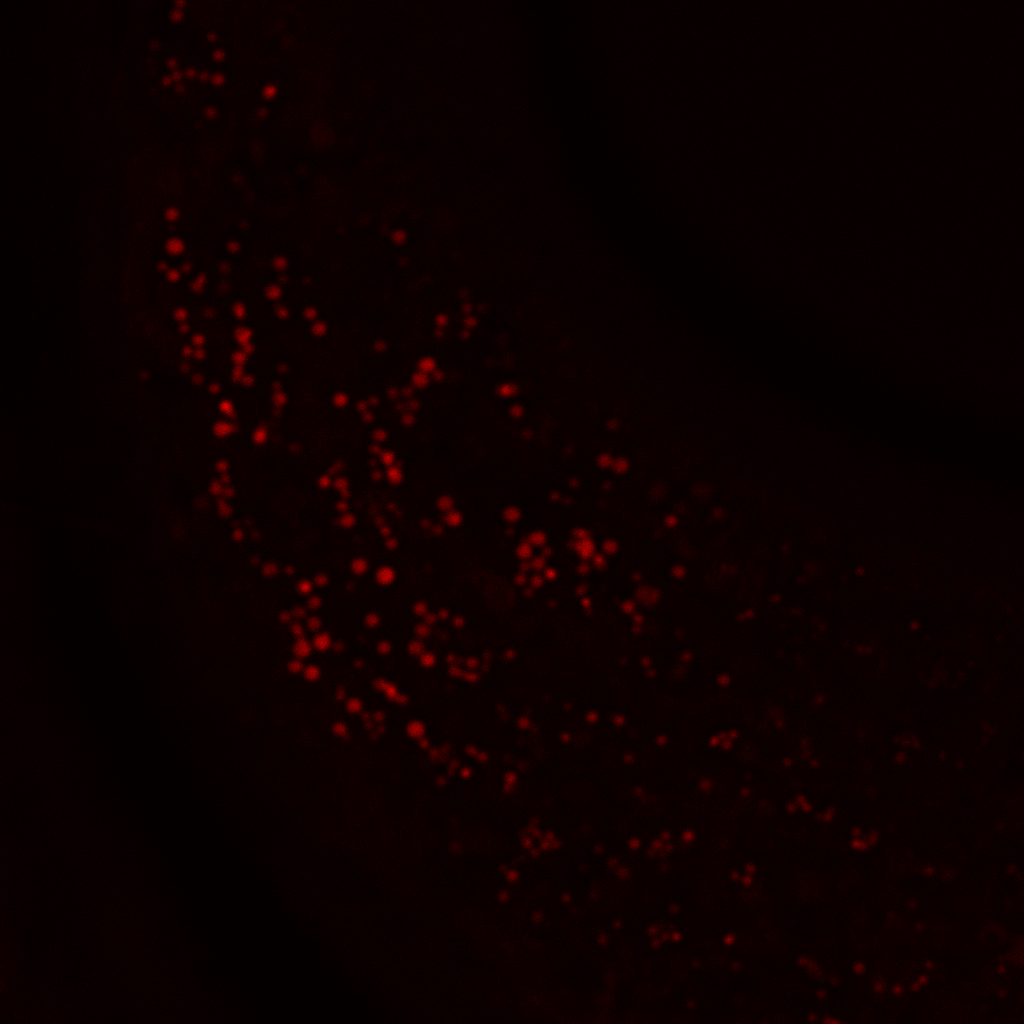

Supplement: Supplemental Information 2 [file peerj-03-1213-s008.zip › Adult/WT Tail 4.png]

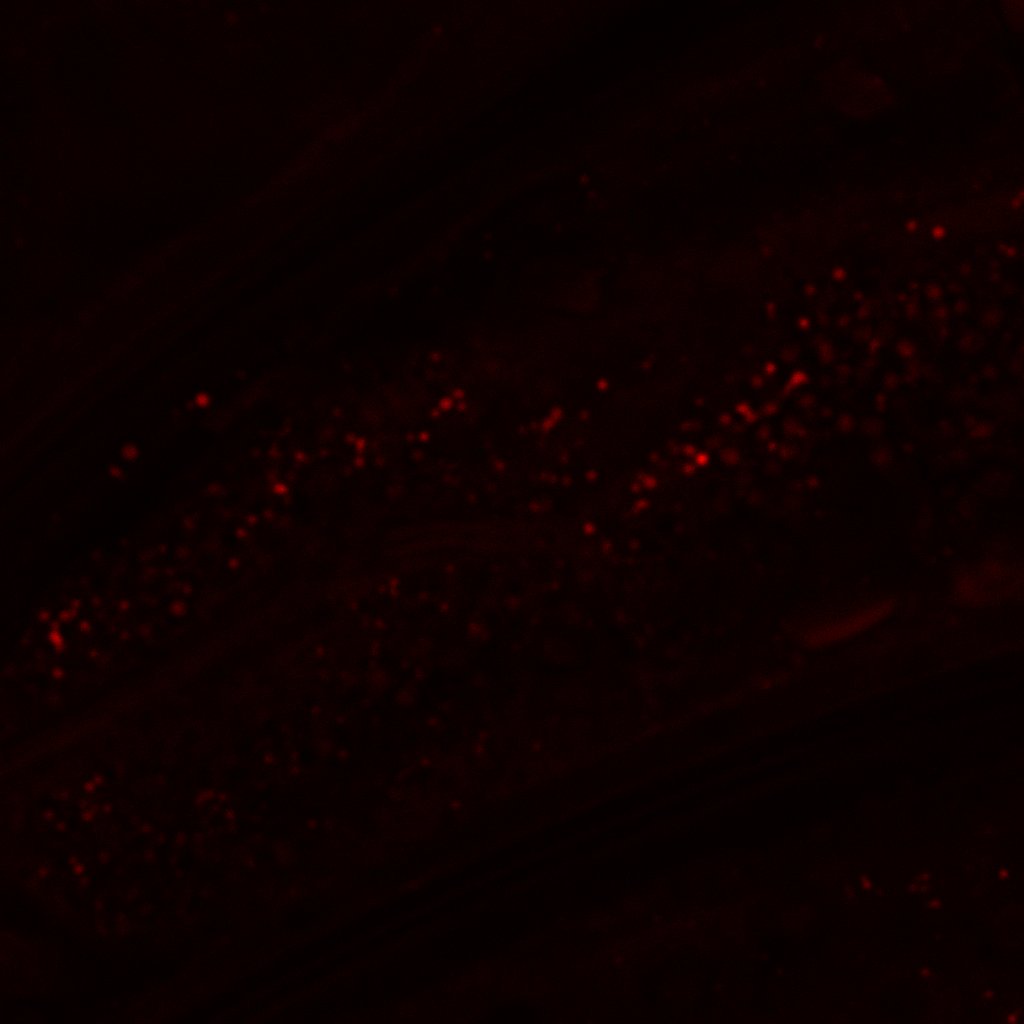

Supplement: Supplemental Information 2 [file peerj-03-1213-s008.zip › Adult/MUT Tail 3.png]

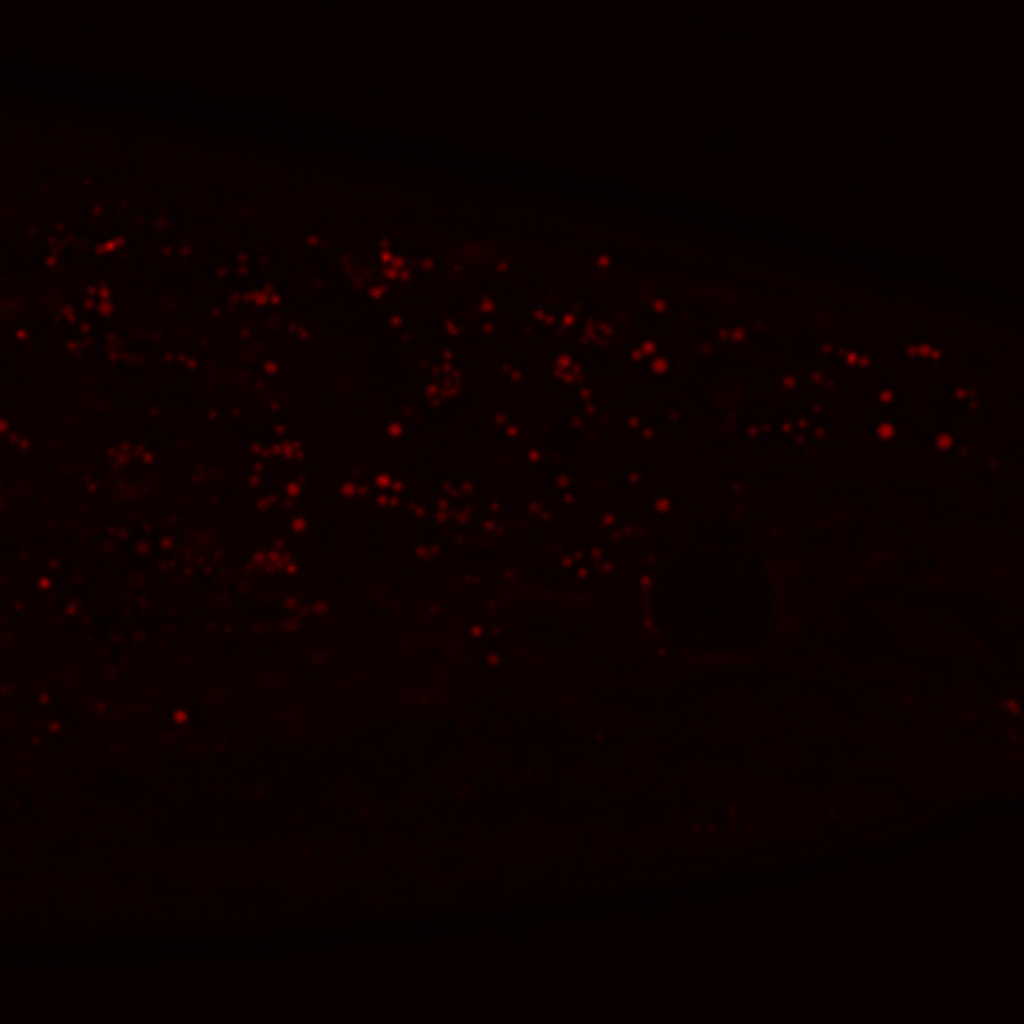

Supplement: Supplemental Information 2 [file peerj-03-1213-s008.zip › Adult/WT Tail 5.png]

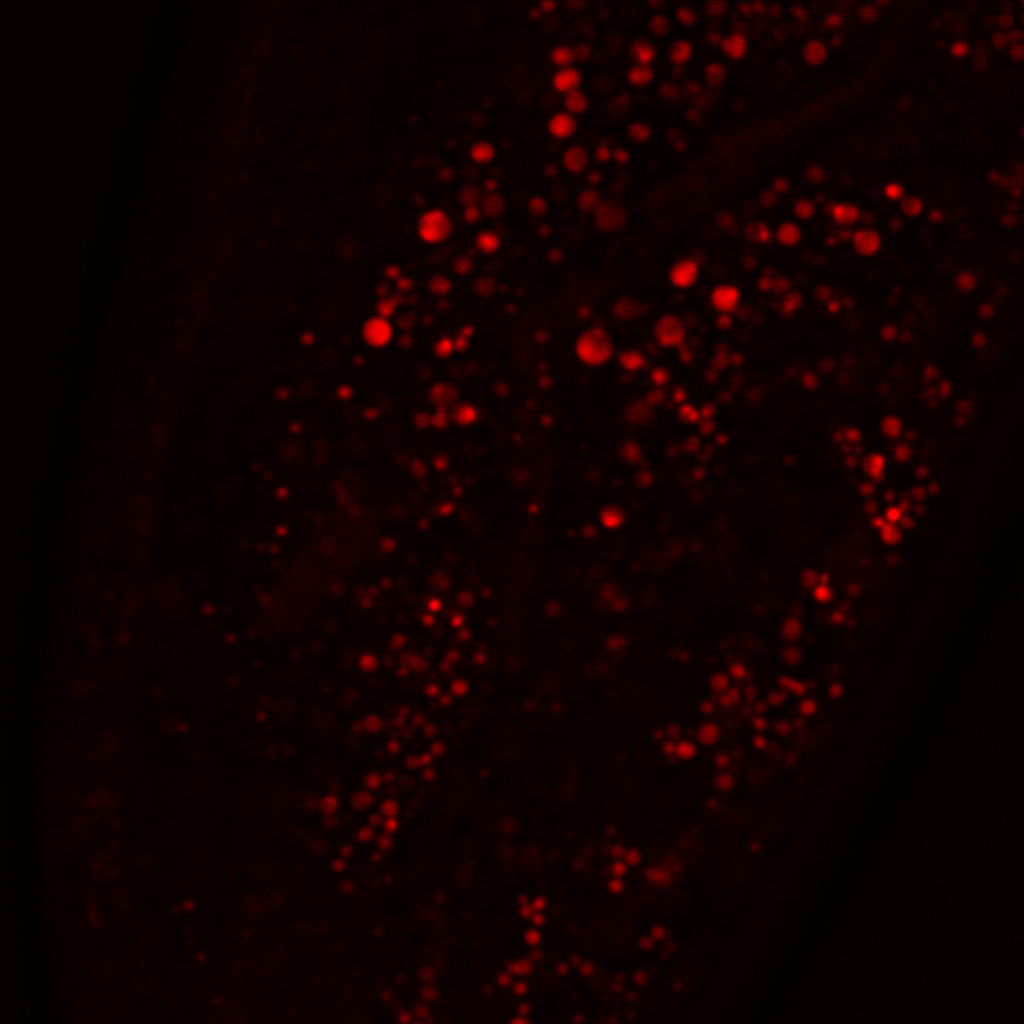

Supplement: Supplemental Information 2 [file peerj-03-1213-s008.zip › Adult/WT Tail 2.png]

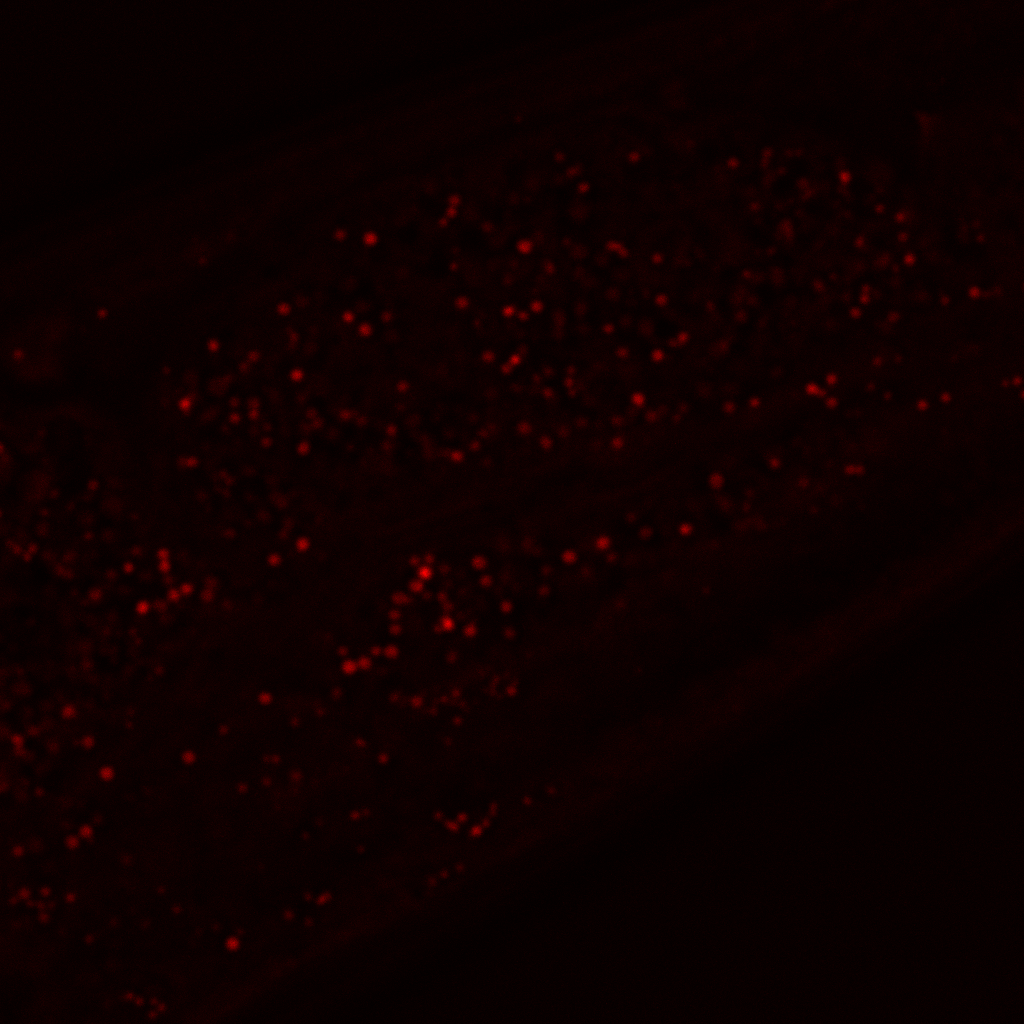

Supplement: Supplemental Information 2 [file peerj-03-1213-s008.zip › Adult/MUT Tail 4.png]

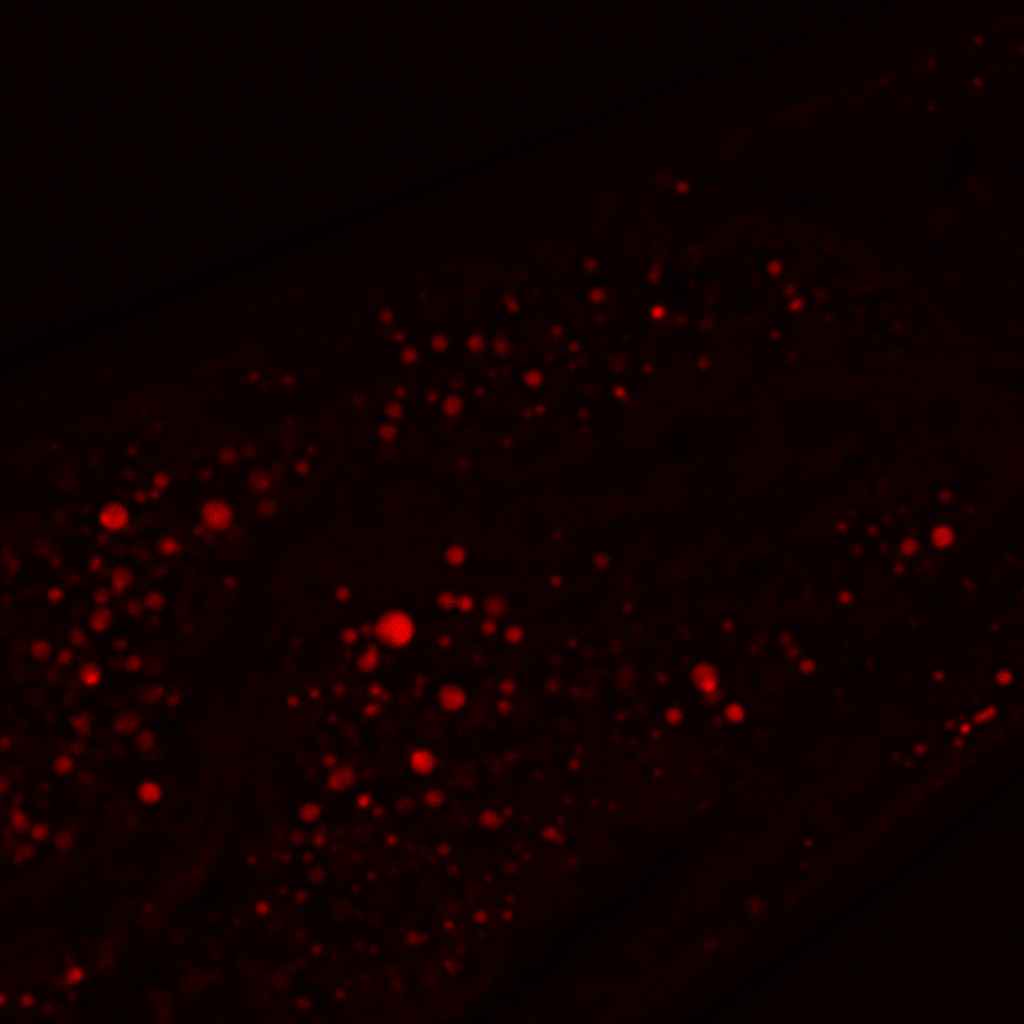

Supplement: Supplemental Information 2 [file peerj-03-1213-s008.zip › Adult/WT Tail 3.png]

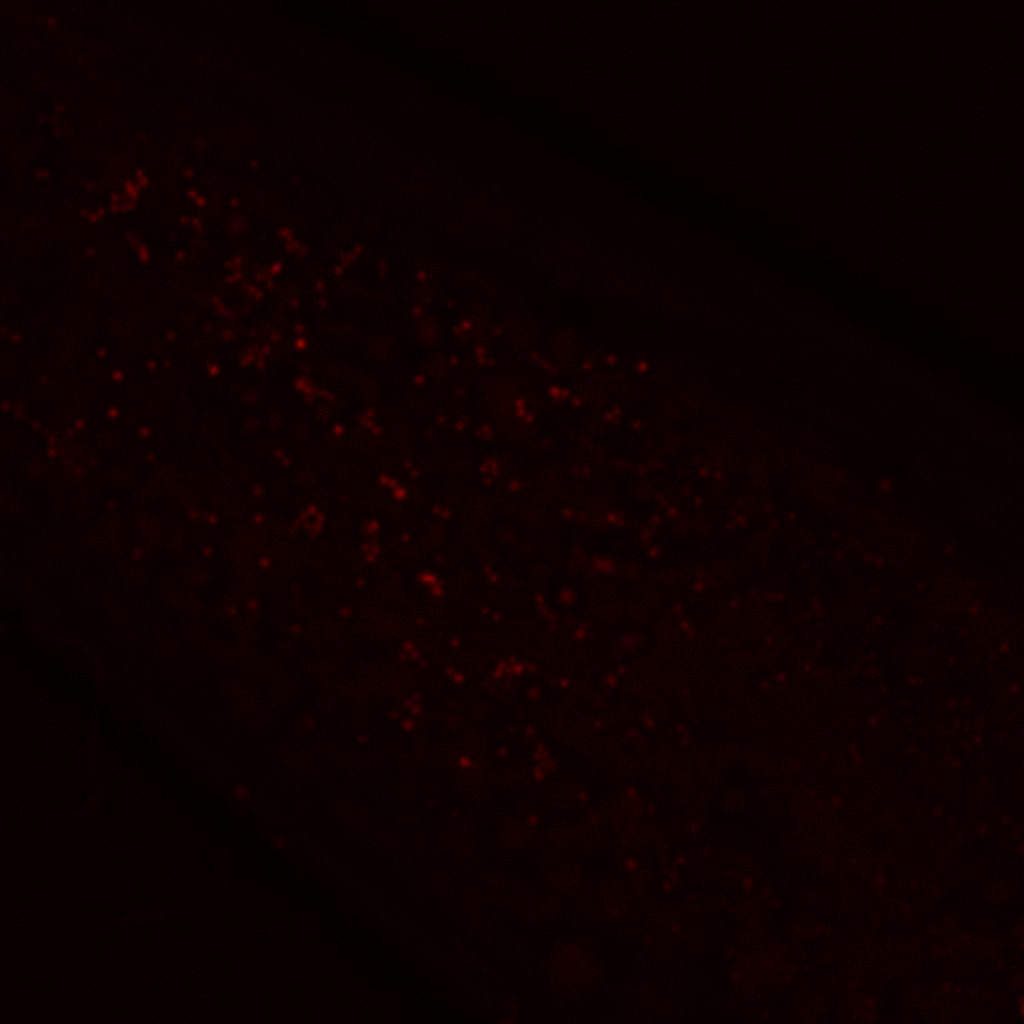

Supplement: Supplemental Information 2 [file peerj-03-1213-s008.zip › Adult/MUT Tail 5.png]
