# Supplementary material for: Perilipin-related protein regulates lipid metabolism in C. elegans
Source: PeerJ. 2015 Sep 1;3:e1213. doi: 10.7717/peerj.1213 (PMC4562238; doi:10.7717/peerj.1213)
Supplement: Supplemental Information 1 [file peerj-03-1213-s006.doc]

# Description of Image Analysis for CARS microscopy

Single focal planes (containing the highest number of CARS positive structures) from stacks of representative embryos and adult hermaphrodites were selected and analyzed using ImageJ computer program. We have provided these selected images as supplement (CARS_QUANTIFICATION-2.ZIP).

All image files are named and the images for embryos are marked with labels. Each label represents the field chosen for analysis for embryos. Each file name and label also corresponds to the data set (containing number and area) generated using ImageJ automatic particle analysis tool as described on <http://imagej.net/Particle_Analysis>. Setting used have been described in the methods section.

File marked as CARS_POSITIVE_STRUCTURES_EMBRYOS.XLS contains data for embryos and each embryo data set is labeled as shown in the supplement image files.

File marked CARS_POSITIVE_STRUCTURES_SOMA_ADULT_TISSUES.XLS contains data for adult somatic tissue analysis. Each data set corresponds to the name of the file provided in the supplement.
