# Supplementary material for: Perilipin-related protein regulates lipid metabolism in C. elegans
Source: PeerJ. 2015 Sep 1;3:e1213. doi: 10.7717/peerj.1213 (PMC4562238; doi:10.7717/peerj.1213)
Supplement: Table S1 [file peerj-03-1213-s007.pdf]

CRISPR/Cas9 Oligomers (sgRNA sequence)

Lab No.	Description	Sequence
7993	Universal reverse primer for CRISPR/Cas9 plasmid.	5'-CAAGACATCTCGCAATAGGA-3'
7992	Forward primer for pCK001	5'-CTTATGAAGCAGTCTCTTA GTTTTAGAGCTAGAAATAGCAAG-3'
8078	Forward primer for pCK023	5'-GGATCGCCGAGCTTTCAACC GTTTTAGAGCTAGAAATAGCAAGT-3'

Oligomers used for cloning of W01A8.1 and human perilipins

Lab No.	Description	Sequence
7957	Start of first exon of W01A8.1 gene	5'-ATGACTGACGTCGAGCAGCCAGTA-3'
7958	Reverse primer used with 7957 to amplify RNAi targeting region	5'-TGAGCTGCTCAAGATAATTACGGGC-3'
7953	Forward primer with HindIII site, positioned at ~ 1kb upstream of ATG of *W01A8.1*	5'-CCCAAGCTTGACCTTCAGTATCGGAGAAAATCGC -3'
7954	Reverse primer with BamHI site, positioned at the codon of *W01A8.1a* sequence	5'-CGGGATCCGGCACGATTCCTATACAAGTTTCTTG-3'
7955	Nested A' primer used for SOEing PCR, positioned downstream of oligomer #7953 location	5'-TTATCAACTATTTCGCCGGTCGGA-3'
7956	Fusion PCR primer B for isoform a and c of *W01A8.1* gene	5'-AGTCGACCTGCAGGCATGCAAGCT GGCACGATTCCTATACAAGTTTCTTG-3'
8044	Fusion PCR primer B for isoform b of *W01A8.1* gene	5'-AGTCGACCTGCAGGCATGCAAGCT TTCGGTTTGTTGACGAACCAAAAGG-3'
7986	Forward primer to linearize pPD95.77(NeoR)	5'-GGATCCCCGGGATTGGCC -3'
7987	Reverse primer to linearize pPD95.77(NeoR)	5'-CTTTCTTGCTGCAAACAATCAATTGATAAAATG-'3
8045	Reverse primer for isoform b	5'-TTCGGTTTGTTGACGAACCAAAAGGC-3'
8061	Reverse primer for isoform a/c	5'-GGCACGATTCCTATACAAGTTTCTTGTG-3'
8035	Forward Plin 2 primer with overhang for pPD95.77(NeoR) vector	5'-TATCATTTTATCAATTGATTGTTTGCAGCAAGAAAGATGGC ATCCGTTGCAGTTGATCC-3'
8036	Forward Plin 2 primer with overhang for pPD95.77(NeoR) vector	5'-TACCTTTGGGTCCTTTGGCCAATCCCGGGGATCCATGAGTT TTATGCTCAGATCGCTGGG-3'
7988	Forward Plin 3 primer with overhang for pPD95.77(NeoR) vector	5'-TTTATCAATTGATTGTTTGCAGCAAGAAAGATGTCTGCCGA CGGGGCAGA-3'
7991	Forward Plin 3 primer with overhang for pPD95.77(NeoR) vector	5'-TTTGGGTCCTTTGGCCAATCCCGGGGATCCCTTCTTCTCCTC CGGGGCTTTCTC-3'
8134	Forward PCR primer for amplification of the genomic region including the deleted segment	5'-CGTACAAACGGTATTTTTATATCGCTTTCCAAAATCTTCGTT TC-3'
8135	Reverse PCR primer for amplification of the genomic region including the deleted segment	5'-TAACCTAGGTCGAGGGATTTTGGACTTTTAAGAATGG-3'
8136	Forward nested primer for sequencing of the genomic region including the deleted segment	5'-TTTATTTTCAGGCAATCAATGCATAC-3'
8137	Reverse nested primer for sequencing of the	5'-GGTGATATAGTAGAACAGGTCAAGTCG-3'

	genomic region including the deleted segment	
8140	Forward synthetic screening primer	5'-TTCGGCTAAGAAGCTCGGATCGTCG-3'
8141	Reverse synthetic screening primer	5'-CACATCTTCGGCGTCGGTAAAATTTTTT-3'

Quantitative RT-PCR oligomers and probes

Lab No.	qPCR Primer Set	Probe	Description
8006	5'-ATTGCGTGAGAAGGGACAAAAC-3'	Roche qPCR probe 22	Exon spanning primer set amplifies W01A8.1 transcript
8007	5'-TGTGAAGTTCTTCTTCAAGTTCTCC-3'		
7961	5'-AGTCGGTGTGCGAAGGAGCA-3'	Roche qPCR probe 133	Exon spanning primer set amplifies *ama-1* transcript
7962	5'-GATAGCGACCGCATCCACCT-3'		

The primers are listed in the order that they were used.
